# The μ-opioid receptor gene and smoking initiation and nicotine dependence

**DOI:** 10.1186/1744-9081-2-28

**Published:** 2006-08-04

**Authors:** Lan Zhang, Kenneth S Kendler, Xiangning Chen

**Affiliations:** 1 Virginia Institute of Psychiatric and Behavioral Genetics, Department of Psychiatry, Virginia Commonwealth University, Richmond, VA 23298, USA; 2 Psychiatry Department, West China Hospital, Sichuan University, Chengdu, China

## Abstract

The gene encoding the mu-opioid receptor (OPRM1) is reported to be associated with a range of substance dependence. Experiments in knockout mice indicate that the mu-opioid receptor may mediate reinforcing effects of nicotine. In humans, opioid antagonist naltrexone may reduce the reinforcing effects of tobacco smoking. Additionally, the OPRM1 gene is located in a region showing linkage to nicotine dependence. The OPRM1 is thus a plausible candidate gene for smoking behavior. To investigate whether OPRM1 contributes to the susceptibility of smoking initiation and nicotine dependence, we genotyped 11 SNPs in the gene for 688 Caucasian subjects of lifetime smokers and nonsmokers. Three SNPs showed nominal significance for smoking initiation and one reached significance for nicotine dependence. The global test for three-marker (rs9479757-rs2075572-rs10485057) haplotypes was significant for smoking initiation (*p *= 0.0022). The same three-marker haplotype test was marginal (*p *= 0.0514) for nicotine dependence. These results suggest that OPRM1 may be involved in smoking initiation and nicotine dependence.

## Background

Tobacco use is a leading cause of preventable diseases in the world and causes nearly 5 million tobacco-related deaths annually [1]. According to the World Health Organization, 1.3 billion people are smokers and half of the smokers in the world today will die of smoking related diseases [2]. Nicotine is the primary factor responsible for the addictive behaviors. In last two decades, family, twin and adoption studies have implicated that genetic factors strongly influence the behaviors of tobacco use and nicotine dependence [3,4]. In the review by Sullivan and Kendler, they estimated that genetic factors accounted for 56% of the variance in the liability of smoking initiation (SI) and 67% of the variance for progression to nicotine dependence (ND) [4].

The μ-opioid receptor encoded by the OPRM1 gene is a key factor contributing to drug addiction [5]. The μ-opioid receptor is a major site of action for endogenous opioid peptides and exogenous opioid drugs. More interestingly, some non-opioid substances including nicotine that have other primary sites of action are likely to induce the release of endogenous opioid peptides, and subsequently activate the μ receptor [5]. For instance, experiments have shown that nicotine causes a release of endogenous opioids in the brains of rat and mouse [6]. Recent studies using inbred and knockout mice strongly suggest that the μ-opioid receptor mediates both the positive and negative reinforcing effects of nicotine. Nicotine-induced antinociception, reward effects and dependence are substantially attenuated in mice lacking the μ-opioid receptor [7,8]. This implies that some aspects of the rewarding valence of nicotine require μ opioid receptors. In humans, opioid receptor antagonist naloxone may reduce the relative reinforcing effects of nicotine, thus it has been used as a smoking cessation drug [9-11]. Lerman and colleagues show that a variant in OPRM1 may predict the treatment responses to clinical nicotine replacement therapy. Smokers who have the Asp40 variant in the OPRM1 gene are likely to have a favorable response to the treatment [12]. Interestingly, the OPRM1 gene is located at 6q24-25, about 4 million basepairs from a suggestive linkage peak of nicotine dependence [13]. All these suggest that the OPRM1 is an attractive candidate gene that may influence the smoking behavior in humans.

More than 100 SNPs in the human OPRM1 gene have been identified, some of which have been tested for associations with a range of substance dependence [14-19]. Most studies focus on analyzing the variants in coding regions or 5' UTRs. The Asn40Asp variant or rs1799971 is one of the most studied polymorphisms in the gene. However, the results are not consistent. Of the studies conducted for the gene, many do not have comprehensive coverage. In this study, we use haplotype-tagging approach to select SNPs and carry out association analyses for both individual markers and haplotypes. Our results indicate that a major haplotype may be involved in tobacco smoking.

## Methods and materials

### Subjects

The sample was drawn from two large population-based twin studies of the Virginia Twin Registry. The sampling and ascertainment procedures were described elsewhere [20,21]. In this study, we used a subset of twins of European ancestry and randomly selected one twin from each pair. All the subjects were unrelated. All individuals were assessed with basic smoking history, the Fagerstrom Tolerance Questionnaire (FTQ) [22] and withdrawal symptoms. The FTQ was an eight-item questionnaire (score range 0–11) widely used to evaluate the severity of nicotine dependence. In this study, the non-smokers (NS) were defined as those who never smoked a cigarette up to the time of the assessment. Regular smokers with low ND (Low-ND) were defined as those who smoked at least 5 cigarettes per week for five years and their FTQ scores were between 0–2 at their lifetime maximum tobacco consumption. Regular smokers with high ND (High-ND) were those who smoked for five years or more and had an FTQ score between 7–11. In order to evaluate separately the influence of OPRM1 on SI and ND, two measurements with overlapping but not identical genetic effects [21,23], we used this 3-group design. The selection of subjects was based on this design. To estimate the influence of OPRM1 on SI, we compared the allele and genotype frequencies between the NS and regular smokers (which included both Low- and High-ND subjects). For that of ND, we compared the frequencies between the Low- and High-ND subjects. Smokers with FTQ scores between 2 and 7 were not used in this dichotomized design. Of the 688 subjects used in this study, 244 were NS, 215 were Low-ND and 229 were High-ND.

The buccal epithelial cell samples were collected using standard cytology brushes (Fisher Scientific, Fair Lawn, New Jersey). DNA was isolated from the brushes as reported previously [24]. All individuals provided informed consent for participation in this study. Sixteen unlinked microsatellite markers were genotyped to assess the potential population stratification and no evidence of stratification was found [24].

### SNPs selection and genotyping

Eleven SNPs were chosen from the dbSNP and Celera genomics database with the following criteria: (1) SNPs located in coding or regulatory regions of the gene or reported associated with drug dependence in literature; (2) haplotype-tagged SNPs suggested by HapMap database [25] or SNPbrowser™ Software 3.0 [26] using the default parameters; and (3) SNPs with minor-allele frequency ≥ 0.10 in the dbSNP database. SNP rs1799971 is a non-synonymous variant (Asn40Asp) located in exon 1. Other SNPs are located in intronic regions of OPRM1. Table 1 listed the characteristics of the selected SNPs.

**Table 1:** Marker characteristics

**Marker name**	**Marker ID**	**Distance between SNPs (bp)**	**Polymorphism**	**Minor allele**	**Minor allele frequency**	**HWE p value**	**Function**
rs1799971	1	0	A/G	G	0.124	0.0906	Asn40Asp
rs510769	2	1,472	A/G	A	0.267	0.4844	intron
rs524731	3	13,073	A/C	A	0.236	**0.0051**	intron
rs1381376	4	18,166	A/G	A	0.161	0.2753	intron
rs9479757	5	18,086	A/G	A	0.088	0.7725	intron
rs2075572	6	660	C/G	C	0.438	0.6936	intron
rs10485057	7	1,251	A/G	G	0.097	**0.0000**	intron
rs9322447	8	11,065	A/G	A	0.493	0.1692	intron
rs609148	9	6,694	C/T	C	0.260	0.6886	intron
rs648893	10	7,614	C/T	T	0.264	0.2524	intron
rs10485058	11	6,586	A/G	G	0.102	0.0679	intron

Genotyping was performed with the TaqMan genotyping method [27]. Briefly, the PCRs were conducted with 384-well microplates. To ensure the quality of genotyping, negative control samples were included in each plate. The PCRs were performed with 5 ng of genomic DNA, 0.25 μl of TaqMan assay mix (Applied Biosystems, Inc., Foster City, CA, USA) and 2.5 μl of TaqMan universal PCR master mix in a total reaction volume of 5 μl. After activating the polymerase and denaturizing DNA by heating at 95°C for 10 minutes, 40 cycles of 92°C for 15 seconds and 55°C for 1 minute were performed. After the reaction, the fluorescence intensities of reporter 1 and 2 (reporter 1:VIC, excitation = 520 ± 10 nm, emission = 550 ± 10 nm; reporter 2: FAM, excitation = 490 ± 10 nm, emission = 510 ± 10 nm) were measured by the Analyst fluorescence plate reader (LJL Biosytems, Sunnyvale, CA). Based on the ratio of fluorescence intensities, genotypes were scored by a Euclidean clustering algorithm developed in our laboratory [28].

### Statistical analysis

To distinguish the effects of the OPRM1 gene on SI and ND, we made two dichotomous comparisons. For the effect on SI, we compared the genotype and allele frequencies of NS with that of regular smokers including both the Low-ND and High-ND groups. For the effect on ND, we compared the genotype and allele frequencies of the Low-ND group with that of the High-ND group. For individual SNP association analyses, genotype and allele frequencies in the three groups were compared by χ^2 ^test or Fisher's exact test when the expected counts were less than 5 in any cell of the contingency table. Pairwise linkage disequilibrium (LD) was estimated for all subjects by the Haploview 3.2 software [29]. Multi-marker haplotype analyses were conducted with the COCAPHASE module of the UNPHASED program [30].

## Results

In this study, we genotyped 11 SNPs in the OPRM1 gene. Nine of the 11 makers were in the Hardy-Weinberg Equilibrium (HWE). Two SNPs (rs524731 and rs10485057) showed significant deviation from the HWE, Table 1. When HWE was examined separately for the 3 subject groups, none of the 3 groups was significantly deviated from HWE for rs524731, the *P *values were 0.182, 0.616 and 0.079 for the NS, Low-ND and High-ND groups respectively. For rs10485057, the HWE deviation was caused largely by the High-ND group, the *P *values for the NS, Low-ND and High-ND were 0.069, 0.979 and 0.000 respectively. The average scoring rate for the 11 SNPs was 95.6% (94.2–98.7%).

Table 2 summarized the results of allelic and genotypic association analyses. Three SNPs (rs2075572, rs10485057 and rs10485058) showed significant differences (*P *= 0.036, 0.012 and 0.033, respectively) in genotype frequencies between the non-smokers and regular smokers. One SNP (rs2075572) showed significant difference in allele frequency between the non-smokers and regular smokers (*P *= 0.016). For ND, only one SNP (rs10485057) reached nominal significance (*P *= 0.0297). The non-synonymous marker, rs1799971, was not significant for SI or ND.

**Table 2:** Single marker association analyses

	**Number of genotypes in the three groups**									**Genotypic**		**Allelic**	
	
	**NS**			**Low-ND**			**High-ND**			**SI**	**ND**	**SI**	**ND**
	
**Marker name**	**11**	**12**	**22**	**11**	**12**	**22**	**11**	**12**	**22**	***P*-Value**	***P*-Value**	***P*-Value**	***P*-Value**
rs1799971	187	46	5	161	49	3	173	41	7	0.891	0.273	0.628	0.836
rs510769	130	82	11	121	74	9	133	73	9	0.828	0.867	0.337	0.940
rs524731	141	78	17	107	63	12	129	64	16	0.988	0.681	0.895	0.758
rs1381376	168	65	7	147	62	5	161	60	4	0.278	0.617	0.806	0.489
rs9479757	195	40	2	169	37	2	190	31	2	0.930	0.539	0.776	0.299
rs2075572	83	121	33	54	102	38	65	101	51	**0.036**	0.441	**0.016**	0.797
rs10485057	196	40	2	170	31	5	192	19	13	**0.012**	**0.030**	0.655	0.964
rs9322447	62	125	43	44	116	48	53	103	59	0.135	0.269	0.067	0.900
rs609148	131	86	14	112	86	11	113	85	17	0.727	0.510	0.409	0.546
rs648893	134	87	14	105	87	10	110	92	16	0.343	0.597	0.233	0.526
rs10485058	164	60	3	154	45	7	177	35	7	**0.033**	0.293	0.354	0.170

Pairwise LD estimates were listed in Table 3, with D' listed below the diagonal and r^2 ^above the diagonal. These analyses indicated that there was only one LD block, covering markers 6–11. From markers 1 to 4, most pairwise LDs were low. However, several markers, i.e., 1, 3 and 5, or rs1799971, rs524731 and rs9479757, were in high LD with markers 6–11 individually. Based on this LD structure, we conducted multi-marker haplotype analyses using only the markers showing substantial pairwise LDs. The markers included in these analyses were markers 1, 3, 5, and 6 to 11. Of these analyses, several combinations reached or approached nominal significance. The most significant combination was combination 5-6-7 or rs9479757-rs2075527-rs10485057, with global *P *values of 0.0021 and 0.0514 for SI and ND respectively, Table 4. In this combination, there were two haplotypes contributing to elevated risks to tobacco smoking: Haplotype 1-2-1, or G-C-A, had a frequency of 0.363 in the regular smokers and that of 0.307 in the non-smokers (*P *= 0.0373), and haplotype 1-2-2, or G-C-G, was observed only in the regular smokers (frequency = 0.013, *P *= 0.0023). For ND, only the minor haplotype 1-2-2 was significant (the frequency in the High-ND group was 0.022, that in the Low-ND group was 0.002, *P *= 0.010). By comparing the patterns of association, it was clear that haplotype 1-2-2 associated with both SI and ND, while haplotype 1-2-1 was associated only with SI. The most abundant haplotype, 1-1-1 for combination 5-6-7 was significantly underrepresented in the regular smokers (*P *= 0.0167). Combination 1-3-5-6-7 produced similar results as that of combination 5-6-7. The *P *values for the global and individual haplotype tests were all comparable. These results indicated that the major risk haplotype defined by markers 5–7 extended to marker 1 in exon 1, the non-synonymous Asn40Asp polymorphism. This risk haplotype carried the A (Asn) allele.

**Table 3:** Pairwise LD of the typed SNPs*

	rs1799971	rs510769	rs524731	rs1381376	rs9479757	rs2075572	rs10485057	rs9322447	rs609148	rs648893	rs10485058
rs1799971		0.00	0.02	0.00	0.00	0.08	0.01	0.10	0.05	0.05	0.02
rs510769	0.03		0.00	0.16	0.00	0.00	0.00	0.01	0.00	0.00	0.00
rs524731	0.59	0.01		0.00	0.28	0.01	0.22	0.01	0.07	0.06	0.02
rs1381376	0.15	0.73	0.01		0.00	0.01	0.01	0.02	0.00	0.00	0.01
rs9479757	0.55	0.20	**0.93**	0.22		0.11	0.84	0.09	0.03	0.03	0.00
rs2075572	**0.86**	0.12	0.18	0.10	**0.91**		0.10	0.79	0.43	0.41	0.11
rs10485057	0.62	0.13	**0.80**	0.30	**0.98**	**0.83**		0.09	0.02	0.01	0.01
rs9322447	**0.86**	0.17	0.16	0.13	**0.94**	**0.98**	**0.86**		0.36	0.35	0.14
rs609148	**1.00**	0.16	**0.79**	0.01	**1.00**	**0.97**	0.63	**0.99**		0.95	0.04
rs648893	**1.00**	0.08	0.71	0.05	**0.92**	**0.95**	0.56	**0.98**	**0.98**		0.04
rs10485058	**0.88**	0.06	0.57	0.17	0.43	**0.93**	**0.75**	**0.94**	**0.86**	**0.82**	

* Below diagonal, D'; above diagonal r^2^

**Table 4:** Multi-marker haplotype analyses

**Marker Combination**	Global *P *Value*	**Risk Haplotype**	**Case Count**	**Case Frequency**	**Control Count**	**Control Frequency**	**Odds Ratio**	**χ**^**2**^	**Risk Haplotype *P *Value**
Smoking Initiation									

5-6-7	**0.0022**	1-2-1	321.9	0.363	149.6	0.307	1.3	4.34	**0.0373**
		1-2-2	11.4	0.013	0.0	0.000	∞	9.30	**0.0023**
5-6-7-8	**0.0031**	1-2-1-2	318.2	0.358	146.6	0.301	1.3	4.74	**0.0294**
		1-2-2-2	11.7	0.013	0.0	0.000	∞	9.66	**0.0019**
1-3-5-6-7	**0.0035**	1-1-1-2-1	282.2	0.318	124.7	0.256	1.6	4.22	**0.0400**
		1-1-1-2-2	10.16	0.011	0.0	0.000	∞	8.37	**0.0038**

Nicotine Dependence									

5-6-7	0.0514	1-2-2	9.9	0.022	1.2	0.003	8.3	6.61	**0.0102**
5-6-7-8	0.0630	1-2-2-2	10.0	0.026	1.0	0.003	8.8	7.09	**0.0077**

* The empirical *p *values obtained from 10,000 permutations for combinations 5-6-7, 5-6-7-8 and 1-3-5-6-7 for SI were 0.0090, 0.0114 and 0.0259 respectively.

## Discussion

The evidence from animal experiments, treatment response and linkage study suggests that OPRM1 may be a gene contributing to the liability of tobacco smoking and nicotine dependence. In this study, we conduct association analyses to test whether the OPRM1 gene is associated with SI and ND. We use a 3-group design to compare the genotypic and allelic distribution of 11 SNPs between the non-smokers and regular smokers and that between the Low- and High-ND smokers. We find that three SNPs show genotypic associations with SI and only one SNP is associated with ND. In haplotype analyses, a core region covered by 3-marker combination 5-6-7 (rs9479757-rs2075572-rs10485057) reaches global significance for smoking initiation. A major haplotype, 1-2-1 or G-C-A, is overrepresented in the regular smokers as compared with the non-smokers. A minor haplotype for the same marker combination, 1-2-2 or G-C-G, is significantly associated with both SI and ND. Since the frequency of the minor haplotype is relatively low (0.013–0.026), there is a chance that this haplotype may be an artifact of the haplotype reconstruction program. It is well known that haplotypes with low frequencies have much higher error rate. On the other hand, the association of the major haplotype 1-2-1 is likely true. In fact, this haplotype remains significant when we restrict the analyses to those haplotypes observed at least once in our data (frequencies were 0.370 and 0.311 for the smokers and non-smokers, and *P *= 0.04084). This haplotype seems to extend to the first exon, carrying the A allele (Asn) of the Asp40Asn polymorphism.

Several studies have shown that the μ-opioid receptor is involved in the reinforcing effects of drugs. The most extensively studied variation in OPRM1 is Asn40Asp (rs1799171), but the findings are not consistent [31,32]. Asn40Asp is an A/G transition at the 118th nucleotide of the coding sequence, causing an amino acid change at position 40 from asparagine (Asn) to aspartate (Asp). This change leads to the loss of a putative N-glycosylation site in the extracellular region. The Asp40 allele is associated with higher β-endorphin affinity, lower blood cortisol levels and higher aggressive threat scores as compared to the Asn40 allele. A recent meta-analysis including 28 distinct samples and over 8000 subjects concludes that this polymorphism does not increase the risk of substance dependence [33]. In our study, this polymorphism itself is not significant. However, we do notice that this polymorphism is in high LD with the core markers defining the associations with both SI and ND. Marker combination 1-3-5-6-7 produces very similar *p *values as that observed with the core markers (see Table 4, comparing combinations 1-3-5-6-7 and 5-6-7). These results suggest that the Asn40Asp mutation is not itself causative. The associations observed in some studies are likely a reflection of its high LD with other causative variant(s) not yet identified. In a recent study of alcohol dependence, this Asn40Asp polymorphism is not associated the phenotypes [19]. Similar to our study, while this polymorphism is partitioned in a different LD block, it does share substantial LD with a risk haplotype found in a separate LD block. Since several markers (rs1799971, rs609148 and rs648893) in the alcohol dependence study are also typed in our study, this allows us to compare the 3 marker haplotype directly. We find that the extended risk haplotype for SI in our sample is 1-1-1-2-1-2-2-2 or A-C-G-C-A-A-C-T for markers 1-3-5-6-7-8-9-10 or rs1799971-rs524731-rs9479757-rs2075572-rs10485057-rs9322447-rs609148-rs648893. Extracting markers 1-9-10 from this extended haplotype, we have 1-2-2 or A-C-T for rs1799971- rs609148-rs648893. This overlaps with one of the risk haplotypes, A-A-C-C-T for markers rs1799971-rs3823010-rs495491-rs609148-rs648893, identified in the alcoholism study. Given the high comorbidity between alcohol drinking and tobacco smoking, this finding is interesting. Because the 5' end of the gene is not in high LD for those markers we typed in our study, we cannot be certain whether the same risk haplotype underlies the risks for both alcoholism and tobacco smoking. Further studies with more markers at the 5' end of the gene are necessary to clarify this issue.

There are several factors contributing to false positives in case control studies, including population stratification, sample size and genotyping error. In our study, we have taken measurements to reduce these risks. We genotyped unlinked microsatellite markers to assess potential stratification and used reasonable sample sizes. We examined HWE for all SNPs. For those two SNPs showing departure from HWE, we checked each subject groups separately. Only one (rs10485057) of these two SNPs involves in the core risk haplotype. For this marker, HWE deviation is caused largely, if not exclusively, by the High-ND smokers. This case-specific HWE deviation, by itself, can be interpreted as an association [34,35]. Multiple testing is another source of false positives. In this study, none of the tests would survive Bonferroni correction.

Since many of these tests are correlated (many SNPs are in substantial LD and there is overlap of markers in multi-marker analyses), Bonferroni correction seems excessive in this case. Based on this rationale, we decide to use permutation tests to obtain empirical p values for the multi-marker haplotype tests. With 10,000 permutations, combinations 5-6-7, 5-6-7-8 and 1-3-5-6-7 remain significant for SI (p = 0.0090, 0.0114 and 0.0259 respectively). Based on these results, we may reasonably conclude that OPRM1 is associated with smoking initiation. Whether OPRM11 is associated with nicotine dependence is less clear. Further studies with independent samples are necessary to resolve these issues.
